# A Case of Collet-Sicard Syndrome Caused by Otitis Externa

**DOI:** 10.7759/cureus.27218

**Published:** 2022-07-25

**Authors:** Sruthi Bonda, Kyaw M Tun, Shadaba Asad

**Affiliations:** 1 Internal Medicine, Kirk Kerkorian School of Medicine at the University of Nevada, Las Vegas, USA; 2 Infectious Diseases, University Medical Center of Southern Nevada, Las Vegas, USA

**Keywords:** collet-sicard, infectious disease, osteomyelitis, cranial nerve palsy, otitis externa

## Abstract

Collet-Sicard syndrome is a unilateral palsy of the lower cranial nerves IX, X, XI, and XII, resulting from lesions at the skull base that affect the jugular foramen and hypoglossal canal. Common causes of the lesions include basilar skull fractures, carotid artery dissections, and malignancy. Infectious and inflammatory etiologies have also been reported. A 63-year-old male with a history of uncontrolled diabetes was admitted for dysphagia, right ear pain, drainage, and right-sided facial droop after recent local trauma and surgical instrumentation of the right ear. Culture of the external auditory canal grew *Pseudomonas aeruginosa*. Triple phase bone scan demonstrated osteomyelitis at the skull base due to complications from otitis externa. The patient's presentation was consistent with Collet-Sicard syndrome, and he was subsequently treated with a six-week course of ciprofloxacin. This patient demonstrates a unique case since his malignant otitis externa spread locally and led to skull base osteomyelitis and subsequently developed Collet-Sicard syndrome. His uncontrolled diabetes likely played a role in his disease progression.

## Introduction

Collet-Sicard syndrome is a unilateral palsy of the lower cranial nerves IX, X, XI, and XII, resulting from lesions at the skull base that affect the jugular foramen and hypoglossal canal [1]. It was first described in 1915 in a World War I soldier whose radiographic studies showed metallic fragments of a bullet at the location of the lesions. The lesions are most commonly due to basilar skull fractures and carotid artery dissections. Malignant causes are also common, such as parotid tumors, glomus jugulare tumors, schwannomas of the hypoglossal nerve, tumors of the skull base, and metastases. Other causes include jugular vein phlebitis and iatrogenic causes such as internal jugular vein catheterization, cerebral angiography, cardiac surgery, and cerebral vessel clamping [2]. While infectious and inflammatory causes are rarer, there have been reported cases of polyarteritis nodosa, Trousseau syndrome, otitis media, Lyme disease, and varicella-zoster causing Collet-Sicard syndrome [2]. There has only been one reported case of skull base osteomyelitis resulting in Collet-Sicard syndrome prior to our case.

## Case presentation

The patient is a 63-year-old male with a past medical history of uncontrolled type 2 diabetes (hemoglobin A1c 11.2%), who was admitted for three days of worsening dysphagia and four months of right ear pain and discharge. He reported that the ear pain and drainage began after he aggressively cleaned his ear with a Q-tip four months prior. Soon after, he underwent an in-office surgical debridement with an otolaryngologist and received a course of azithromycin. Approximately three weeks later, he developed numbness of the right side of the face, difficulty closing the right eye, and drooling from the right side of the mouth, and was treated with corticosteroids which temporarily relieved these Bell’s palsy-type symptoms. However, two months after treatment with corticosteroids, the symptoms recurred. In addition, he developed a raspy voice that persisted to the time of his hospital admission. His dysphagia was characterized by the inability to swallow puree-consistency food or water.

**Figure 1:**
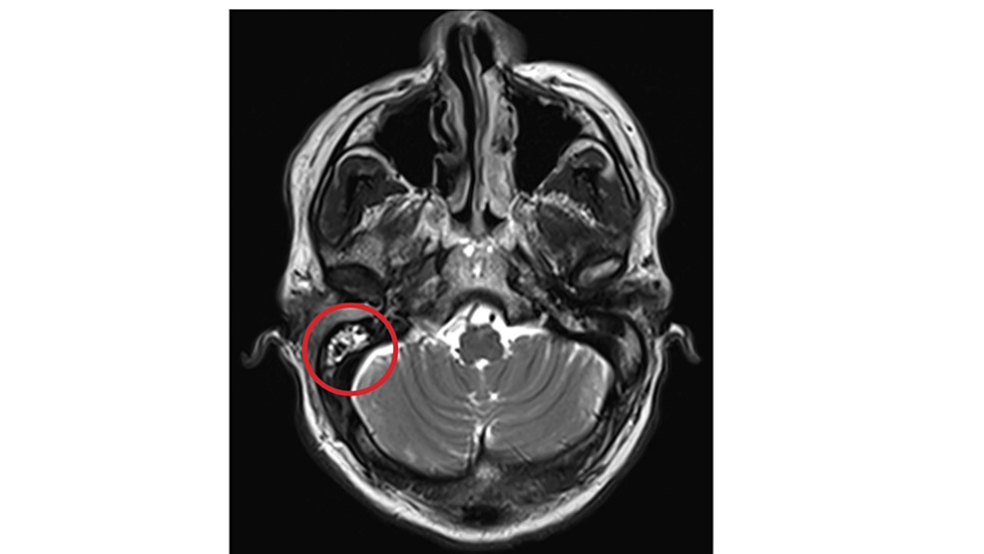
MRI showing a moderate right mastoid effusion

**Figure 2:**
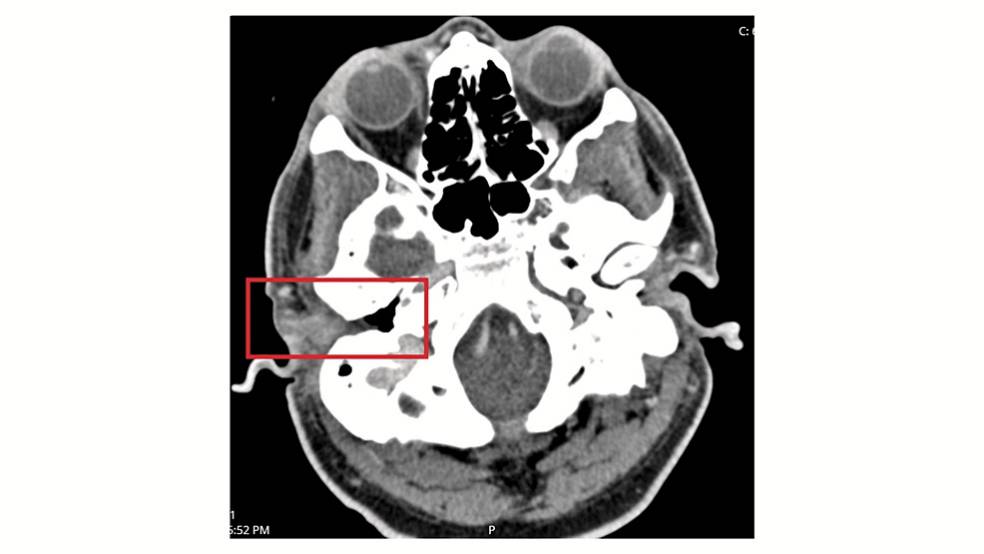
CT scan showing was near-complete opacification of the right external auditory canal

**Figure 3:**
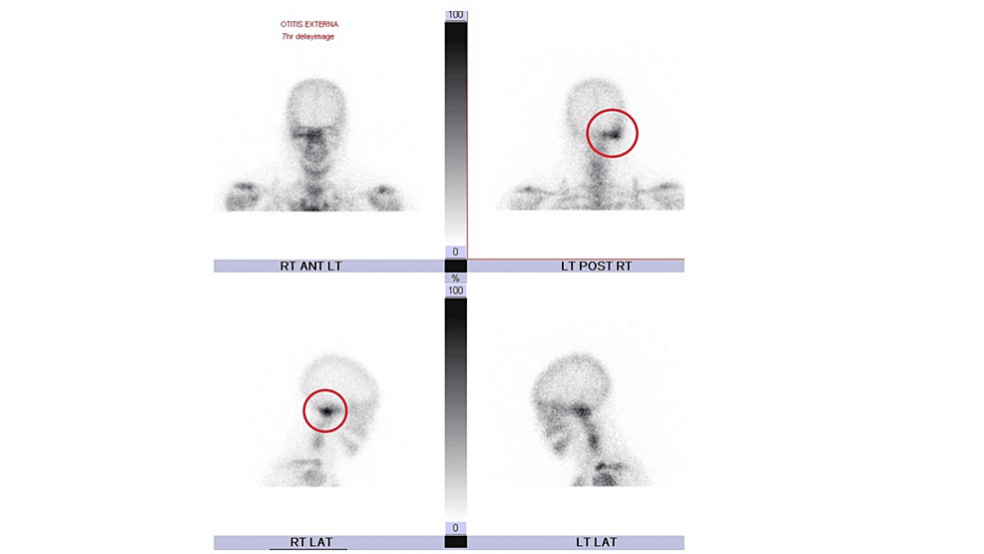
Triple phase bone scan showing an abnormality at the right lateral skull base

On presentation, the patient had a noticeable right-sided facial droop and the inability to raise the right eyebrow and close the right eyelid. He also had right-sided tongue deviation and erythema and edema of both the right external auditory canal and tympanic membrane. He underwent a modified barium swallow study, which demonstrated poor peristalsis, vallecula and piriform pooling, and silent aspiration. Laboratory studies were remarkable for leukocytosis at 10.39 k/mm^3^(reference: 3.10-10.20), ^ ^elevated erythrocyte sedimentation rate and C-reactive protein at 88 mm/hr (reference: 0-10), and 27.0 mg/L (reference: ≤3), respectively. Infectious disease labs, including herpes simplex virus (HSV), human immunodeficiency virus (HIV), *Borrelia burgdorferi*, and varicella-zoster virus, were negative. Culture of the external auditory canal grew pan-sensitive *Pseudomonas aeruginosa*. Biopsy of the right external auditory canal demonstrated granulation tissue with mixed acute and chronic inflammatory infiltrates. There was no evidence of cellular atypia or malignancy. Computed tomography (CT) imaging revealed no acute pathology intracranially or in the internal auditory canal. However, there was near-complete opacification of the right external auditory canal, effacement of the right tympanic membrane, and enhancement around the right carotid space and the right parapharyngeal muscles. Magnetic resonance imaging (MRI) of the brain showed a moderate right mastoid effusion (Figure 1). A magnetic resonance angiogram of the cervical arterial vasculature showed no abnormalities. CT imaging of the cervical soft tissue showed enhancement extending from the right external auditory canal into the right carotid space, suggestive of an inflammatory process (Figure 2). Triple phase bone scan further highlighted an abnormality in the right lateral skull base (Figure 3). Based on the imaging findings and elevated inflammatory markers, a diagnosis of osteomyelitis of the skull base was made.

A lumbar puncture was performed but was not remarkable for any infectious etiology or other acute causes. It was determined that the patient’s neurological symptoms were due to his right-sided skull base osteomyelitis, which was in turn a complication from otitis externa. The patient was started on intravenous ciprofloxacin and then discharged on oral ciprofloxacin for a total antibiotic duration of six weeks. A percutaneous gastrostomy tube was placed due to the aforementioned dysphagia prior to discharge. When the patient was re-evaluated two months after discharge, his neurological and malignant otitis externa symptoms had resolved. The patient was also able to eat and drink orally, and the gastrostomy tube was subsequently removed.

## Discussion

This patient has a unique disease course, given that there has only been one similar case published recently. His symptoms began after trauma to the external auditory canal and tympanic membrane, which was possibly exacerbated by the outpatient debridement procedure that he underwent. This chronic perforation and effusion resulted in otitis externa, the causative pathogen being *Pseudomonas aeruginosa*. Over time, this infection spread locally and led to right-sided lateral skull base osteomyelitis. The patient's uncontrolled diabetes likely also played a role in his infectious disease progression. This infectious process also involved the right internal auditory canal and the cerebellopontine angle. This explains the patient's right facial droop since cranial nerve VII would be affected. Internal carotid artery stenosis has been reported in other cases of infectious Collet-Sicard syndrome [3] but was unlikely the culprit in our patient's case. This patient also exhibited palsies of cranial nerves IX, X, XI, and XII, due to osteomyelitis of the skull base that involved the jugular foramen and hypoglossal canal. This patient is unique in that he developed a skull-based infection as a result of malignant otitis externa, which commonly originates from the external ear canal [4]. Previously reported cases of Collet-Sicard syndrome attribute the infectious origin to otitis media. Sibai et al. describe a case of a patient who developed a cranial-based infection and mass effect due to incomplete treatment of otitis media [4]. Blazina et al. also describe a patient who developed Collet-Sicard syndrome due to chronic otitis media from *Pseudomonas aeruginosa* [5]. There has only been one documented case of Collet-Sicard syndrome from malignant otitis externa, reported by Climans et al., where the causative pathogen was also *Pseudomonas aeruginosa* [6]. 

Our patient was tested for other causes of infectious disease, including HSV, HIV, *Borrelia burgdorferi*, and varicella-zoster virus, since there have been reports of these conditions also causing cranial nerve palsies. Kahane et al. describe a patient with jugular foramen syndrome with palatolaryngeal herpetic eruption, aseptic meningitis, and a high level of serum antibody to varicella-zoster [7]. Kondo et al. report another case of a patient with herpes zoster infection in the form of meningoencephalitis that resulted in palsies of cranial nerves IX to XI [8]. Miyazaki et al. report a case of a patient with hoarseness and dysphagia from pharyngeal and vocal cord palsies from Ramsay Hunt syndrome [9]. Another case report describes a young patient with Lyme disease who developed lower cranial nerve palsies [10]. Our patient was negative for the infectious diseases that are more commonly associated with cranial nerve palsies.

## Conclusions

As demonstrated by our case, malignant otitis externa can result in skull base osteomyelitis and cranial nerve palsies. This infectious cause is important to consider because the patient’s neurological symptoms can easily be confused for cerebrovascular disease or other more common etiologies, which might delay the necessary treatment. It is also important to be aware of Collet-Sicard syndrome and other cranial nerve palsies as potential complications from malignant otitis externa and skull base osteomyelitis, especially in older patients with poorly controlled diabetes.

## References

[1] Gutiérrez Ríos R, Castrillo Sanz A, Gil Polo C, Zamora García MI, Morollón Sánchez-Mateos N, Mendoza Rodríguez A (2015). Collet-Sicard syndrome. Neurologia.

[2] Handley TP, Miah MS, Majumdar S, Hussain SS (2010). Collet-Sicard syndrome from thrombosis of the sigmoid-jugular complex: a case report and review of the literature. Int J Otolaryngol.

[3] Mutsukura K, Tsuboi Y, Imamura A, Fujiki F, Yamada T (2004). [Garcin syndrome in a patient with rhinocerebral mucormycosis]. No To Shinkei.

[4] Sibai TA, Ben-Galim PJ, Eicher SA, Reitman CA (2009). Infectious Collet-Sicard syndrome in the differential diagnosis of cerebrovascular accident: a case of head-to-neck dissociation with skull-based osteomyelitis. Spine J.

[5] Blažina K, Bazadona D, Relja M, Gardijan D (2016). Collet-Sicard syndrome as a complication of chronic middle ear infection. Clin Case Rep Rev 1.

[6] Climans SA, Melanson M, Desai JA (2013). A case of Collet-Sicard syndrome caused by necrotizing otitis externa. Can J Neurol Sci.

[7] Kahane P, De Saint Victor JF, Besson G, Hommel M, Perret J (1993). [Jugular foramen syndrome caused by herpes zoster]. Rev Neurol (Paris).

[8] Kondo M, Hokezu Y, Nagai M, Mori T, Nagamatsu K (1994). [A case of herpes zoster meningoencephalitis followed by involvement of cranial nerves IX, X, XI]. Rinsho Shinkeigaku.

[9] Miyazaki Y, Tajima Y, Sudo K, Matsumoto A, Kikuchi S, Tashiro K (2002). [A case of Ramsay Hunt syndrome initiated with hoarseness and dysphagia: consideration on spreading mechanisms of cranial neuropathy]. Rinsho Shinkeigaku.

[10] Piché-Renaud PP, Branson H, Yeh EA, Morris SK (2018). Lyme disease presenting with multiple cranial neuropathies on MRI. IDCases.

